# Zingerone Targets Status Epilepticus by Blocking Hippocampal Neurodegeneration via Regulation of Redox Imbalance, Inflammation and Apoptosis

**DOI:** 10.3390/ph14020146

**Published:** 2021-02-11

**Authors:** Summya Rashid, Adil Farooq Wali, Shahzada Mudasir Rashid, Rana M. Alsaffar, Ajaz Ahmad, Basit L. Jan, Bilal Ahmad Paray, Saeed M. A. Alqahtani, Azher Arafah, Muneeb U. Rehman

**Affiliations:** 1 Department of Pharmacology & Toxicology, College of Pharmacy Girls Section, Prince Sattam Bin Abdulaziz University, P.O. Box-173, Al-Kharj 11942, Saudi Arabia or s.abdulrashid@psau.edu.sa (S.R.); r.alsaffar@psau.edu.sa (R.M.A.); 2 Department of Pharmaceutical Chemistry, RAK College of Pharmaceutical Sciences, RAK Medical and Health Sciences University, Ras Al Khaimah P.O. Box 11172, United Arab Emirates; 3 Division of Veterinary Biochemistry, Faculty of Veterinary Science and Animal Husbandry, SKUAST-Kashmir, Alustang, Shuhama 190006, India; mudasir@skaustkashmir.ac.in; 4 Department of Clinical Pharmacy, College of Pharmacy, King Saud University, P.O. Box-2457, Riyadh 11451, Saudi Arabia; aajaz@ksu.edu.sa (A.A.); bjan@ksu.edu.sa (B.L.J.); aazher@ksu.edu.sa (A.A.); 5 Department of Zoology, College of Science, King Saud University, P.O. Box-2457, Riyadh 11451, Saudi Arabia; bparay@ksu.edu.sa; 6 Department of Pharmacology & Toxicology, College of Pharmacy, King Saud University, P.O. Box-2457, Riyadh 11451, Saudi Arabia; 439106181@student.ksu.edu.sa

**Keywords:** status epilepticus, zingerone, inflammation, apoptosis, caspase-3, Bcl-2, redox status, histology, cognition, NF-κB

## Abstract

Epilepsy is an intricate neurological disease where the neurons are severely affected, leading to the mortality of millions worldwide. Status epilepticus (SE), induced by lithium chloride (LiCl) and pilocarpine, is the most accepted model for epilepsy. The current work aims to unravel the mechanisms underlying the anti-epileptic efficacy of zingerone (an active ingredient of ginger), which has beneficial pharmacological activities on seizure-induced behavioral, histological, neurochemical, and molecular patterns in mice. Zingerone restored cognitive function by diminishing seizure activity, escape latency, and subsequent hippocampal damage manifested in histology. Seizures are associated with local inflammation, redox imbalance, and neural loss, confirmed by the present study of SE, and was attenuated by zingerone treatment. Nuclear factor-kappa B and its downstream signaling molecules (TNF-α, IL-1β, IL-6, NO, MPO) were activated in the LiCl-and-pilocarpine-induced group leading to inflammatory signaling, which was substantially ameliorated by zingerone treatment. The intrinsic apoptotic process was triggered subsequent to SE, as demonstrated by augmentation of cleaved caspase-3, downregulation of Bcl-2. However, zingerone treatment downregulated caspase-3 and upregulated Bcl-2, increasing cell survival and decreasing hippocampal neural death, deciphering involvement of apoptosis in SE. Therefore, zingerone plays an essential role in neuroprotection, probably by precluding oxidative stress, inflammation, and obstructing the mitochondrial pathway of apoptosis.

## 1. Introduction

Epilepsy is the fourth most widespread, chronic, non-communicable illness of the brain affecting people of all ages. Roughly 50 million individuals are afflicted by this disease worldwide; the rate of active epilepsy population at a given time is between 4 to 10 persons per 1000 people [1]. The major symptoms are recurrent seizures, which could involve a part of the body or the whole body, sometimes together with the loss of consciousness and bladder and bowel control functions [2]. Currently, several distinguished forms of epilepsy are known, with temporal lope epilepsy (TLE) being the commonest and resistant to treatment [3]. TLE is typified by loss of neurons and hippocampal reactive gliosis, entorhinal cortex, or amygdala of the brain [4]. If not treated or if not responding to medication, some patients can experience protracted seizures, status epilepticus (SE), which is a deadly condition. Its treatment primarily includes the dissolution of seizures as rapidly as possible. The treatment includes antiepileptic drugs or anesthetics, but these drugs are often known to exacerbate morbidities [5].

Reports suggest that systemic injection of pilocarpine, muscarinic receptor agonist produces uninterrupted epileptic seizures, which are comparable with human epilepsy, constituting the well-established model in mice [6]. The mechanism underlying the disease onset and progression is still under debate, but oxidative stress with neuroinflammation, neural death, and abnormal connections between neurons are possible pathophysiological features [7]. Undoubtedly, the brain is predisposed highly to oxidation owing to its large content of lipids and low antioxidant enzymes; free radicals can act as a pathogen for epilepsy. Alteration in reactive oxygen species (ROS), reactive nitrogen species (RNS), and nitric oxide (NO) signaling pathways are associated with oxidative stress [8]. A couple of reactive species has the ability to trigger inflammatory pathways, which in turn exaggerates the overall redox imbalance. Neuroinflammation is crucial in progressing epilepsy, and reports suggest the activation of cytokines and microglia in the epileptic brain [9]. The expression of inflammatory mediators activates different downstream signaling pathways leading to further increase in oxidative stress, and ultimately, all these pathways work together, causing activation of the apoptotic pathways, both intrinsic and extrinsic, leading to activation of effector caspase-3, causing neural loss and integrity [10]. Various reports suggest that dietary supplements with nutritional values can improve the antioxidant content, minimize brain damage, and improve cognitive function [11]. Zingerone (4-[4-Hydroxy-3-methoxyphenyl]-2-butanone), a naturally occurring alkaloid, among active components isolated from ginger, *Zingiber officinale* rhizome [utilized as a spice and a flavoring agent globally]. Its antioxidant and anti-inflammatory potential are reported in the treatment of cancer, diabetes, toxicity, fibrosis, and sepsis [12,13,14,15]. Ginger extracts and its constituents recover learning and retention deficits in amnesia in C57BL/6 mice induced by scopolamine [16]. Zingerone is reported to rapidly cross the blood–brain barrier (BBB) and metabolize in rats and humans easily, achieving decent concentration in blood. It is found to impede deterioration in endogenous antioxidants by radiation and scavenging ROS, and protect brain mitochondria in tellurium toxicity [17,18]. Another report suggested the anti-parkinsonism potential of zingerone due to its potential antioxidant effect of scavenging free radicals and redox ions [19]. In cerebral ischemia, delayed administration of zingerone attenuated the behavioral, histological alterations restoring the overall antioxidant pool [20]. It is also found to be antagonistic against serotonin receptors, thereby acting as a 5-HT3 receptor noncompetitive antagonist [21]. Ethanolic extract of rhizomes of *Zingiber officinale* has been found to have anticonvulsant activity in experimental animals [22]. The above-mentioned studies suggest the preventive role of zingerone on neurological diseases, but until now its effect on pilocarpine-induced epilepsy in mice is unknown. The objective of this current work was to assess the possible pathways involved in the progression of the disease and its prevention by zingerone. We extensively studied inflammatory mediators and markers of inflammation such as NF-κB, Tumor Necrosis Factor Alpha (TNF-α), Interleukin 6 (IL-6), Interleukin 1beta (IL-1β), nitric oxide (NO), myeloperoxidase (MPO); markers of oxidative stress such as reactive oxygen species (ROS), lipid peroxidation; depletion of antioxidant machinery such as reduced glutathione (GSH), glutathione reductase (GR), catalase (CAT), superoxide dismutase (SOD), and histological alterations. We also unraveled intrinsic apoptotic pathway proteins such as cleaved caspase-3 and Bcl2 and their regulation with zingerone treatment. Therefore, the current work aimed to assess the beneficial properties of zingerone on SE in vivo to further illuminate its molecular mechanism involving redox imbalance, inflammation, and neural death.

## 2. Results

### 2.1. Effect of Zingerone and Sodium Valproate on Seizure Activity against LiCl-and-Pilocarpine-induced Epilepsy

Administration of zingerone at 25 and 50 mg/kg b.w dose to mice for 15 days, followed by giving LiCl 15–20 h prior to pilocarpine on the last day of drug treatment for the induction of epilepsy in mice revealed the following scores.

#### 2.1.1. Effect on Latency to Start of Seizures

The latency to seizure onset score revealed a significant (*** *p* < 0.001) rise in mice of the Standard drug group, compared to the score observed in positive control that received LiCl and pilocarpine only. Pre-treatment with zingerone at 25 mg/kg b.w. to mice in group IV demonstrated considerable (* *p* < 0.05) rise in the latency to seizure arrival score while the mice of group V that received 50 mg/kg b.w. of zingerone revealed a more substantial (*** *p* < 0.001) rise in the score (Table 1).

#### 2.1.2. Effect on Percentage Convulsion

The percentage convulsion was significantly lowered (** *p* < 0.01) in mice of the Standard drug group compared to that observed in the positive control (receiving LiCl and pilocarpine only). Pre-treatment with zingerone at 25 mg/kg in group IV showed a noteworthy (* *p* < 0.05) fall in convulsion percentage while group V receiving a higher dose of zingerone (50 mg/kg) witnessed a more substantial (** *p* < 0.01) fall in convulsion percentage (Table 1).

### 2.2. Effect of Zingerone and Sodium Valproate on Escape Latency and Probe Trial of Morris Water Maze

**Figure 1:**
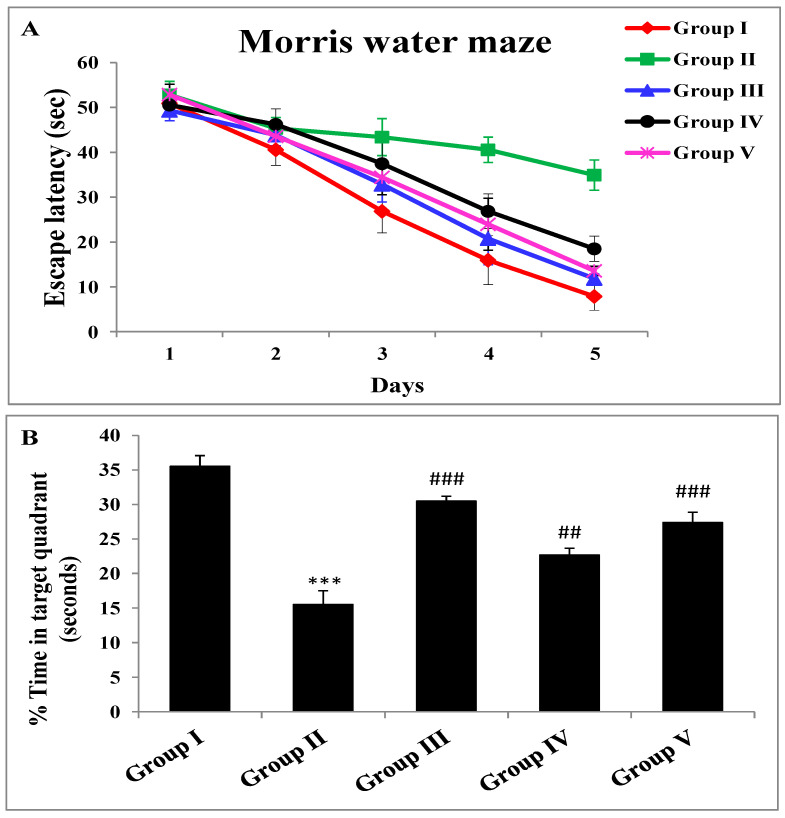
Effect of zingerone on the cognitive function of LiCl-and-pilocarpine-induced SE in mice by Morris water maze. (**A**) Effect on escape latency (sec) on pilocarpine-induced status epilepticus. Results are representative of mean ± SE of fourteen mice per group. In group-II, the escape latency was increased significantly (*** *p* < 0.001) as compared to control group (group I). Treatment with sodium valproate and zingerone (25 and 50 mg/kg b.w.) significantly attenuated escape latency level in group III (^###^ *p* < 0.001), group IV (^##^ *p* < 0.01), and group V (^###^ *p* < 0.001) as compared to group II. Group I: Normal saline (10 mL/kg b.w.), Group II: LiCl (3 mEq/kg b.w.) + pilocarpine (30 mg/kg b.w.), Group III: Sodium Valproate (300 mg/kg b.w.) + LiCl (3 mEq/kg b.w.) + pilocarpine (30 mg/kg b.w.), Group IV: zingerone (25 mg/kg b.w.) + LiCl (3 mEq/kg b.w.) + pilocarpine (30 mg/kg b.w.), Group V: zingerone (50 mg/kg b.w.) + LiCl (3 mEq/kg b.w.) + pilocarpine (30 mg/kg b.w.) (**B**) Effect of zingerone on % time in target quadrant (seconds) on LiCl-and-pilocarpine-induced SE. Results are representative of mean ± SE of eight mice per group. The results that we got are significantly different from pilocarpine group as the main comparison is with pilocarpine group only (^##^ *p* < 0.01 and ^###^ *p* < 0.001). Group I: Normal saline (10 mL/kg b.w.), Group II: LiCl (3 mEq/kg b.w.) + pilocarpine (30 mg/kg b.w.), Group III: Sodium Valproate (300 mg/kg b.w.) + LiCl (3 mEq/kg b.w.) + pilocarpine (30 mg/kg b.w.), Group IV: zingerone (25 mg/kg b.w.) + LiCl (3 mEq/kg b.w.) + pilocarpine (30 mg/kg b.w.), Group V: zingerone (50 mg/kg b.w.) + LiCl (3 mEq/kg b.w.) + pilocarpine (30 mg/kg b.w.).

Escape latency diminished with progress in days in all groups. Group II mice demonstrated substantially (*** *p* < 0.001) greater latency than group I mice. Pretreatment with zingerone at 25 mg/kg exhibited a noteworthy (^##^ *p* < 0.05) decline in the escape latency to find a hidden platform as compared to the group that received LiCl and pilocarpine only (group II). The decrease in escape latency for group III and group V was more substantial (^###^ *p* < 0.001) in comparison with positive control mice (Figure 1A). When the platform was removed, group II devoted less time pointedly (*** *p* < 0.001), owing to forgetfulness in the target quadrant in comparison with group I mice. The percentage of time given in the target quadrant was significantly increased by the pretreatment of zingerone of a lower dose (^##^ *p* < 0.01) and a higher dose (^###^ *p* < 0.001) when contrasted with group II mice. Sodium valproate, the standard antiepileptic drug administered to mice of group III, displayed substantial (^###^ *p* < 0.001) growth in the time being in the target quadrant in comparison to the positive control group (Figure 1B).

### 2.3. Zingerone and Sodium Valproate Effects on Different Biochemical Parameters against LiCl-and-pilocarpine-induced Epilepsy

#### 2.3.1. Lipid Peroxidation (MDA)

We observed a substantial (*** *p* < 0.001) rise in MDA in mice of group II in comparison with negative control mice. A noteworthy (^#^ *p* < 0.05) diminution was observed in group IV mice that had received zingerone (25 mg/kg) while an extremely substantial (^###^ *p* < 0.001) fall in levels of MDA was seen in mice administered zingerone (50 mg/kg) compared to levels in the toxic group that had received LiCl and pilocarpine only (group II). Sodium valproate, the standard antiepileptic drug administered to mice of group III exhibited a substantial (^###^ *p* < 0.001) fall in MDA when compared to group II (Table 2).

#### 2.3.2. Superoxide Dismutase Activity (SOD)

Substantial (*** *p* < 0.001) fall in SOD was detected in mice of positive control when compared with negative control mice. Increase in SOD (^#^ *p* < 0.05) was detected substantially in group IV mice that had received zingerone (25 mg/kg) while more significant (^###^ *p* < 0.001) rise was seen in mice administered zingerone (50 mg/kg) compared to LiCl and pilocarpine only treated group (group II). Sodium valproate, the standard antiepileptic drug administered to mice of group III, displayed a substantial (^###^ *p* < 0.001) rise in SOD in comparison to group II (Table 2).

#### 2.3.3. Catalase Activity (CAT)

A substantial reduction occurred in CAT in mice of group II in comparison to activity in control mice. A marked rise (^#^ *p* < 0.05) in CAT was detected in group IV mice that had received zingerone (25 mg/kg) while a significant (^###^ *p* < 0.001) rise was seen in mice with administered zingerone (50 mg/kg) compared to levels in the group that had received LiCl and pilocarpine only (group II). Sodium valproate, the standard antiepileptic drug administered to mice of group III, showed an extremely substantial (^###^ *p* < 0.001) rise in CAT activity as contrasted to group II mice (Table 2).

#### 2.3.4. Glutathione Reductase Levels (GR)

An extremely significant (*** *p* < 0.001) fall occurred in GR in mice of group II in comparison to levels in control mice (Group I). A non-significant (ns) rise was detected in mice of group IV that had received zingerone (25 mg/kg) while a highly significant (^##^ *p* < 0.01) rise was detected in mice treated with zingerone (50 mg/kg) compared to levels in LiCl and pilocarpine mice (group II). Sodium valproate, the standard antiepileptic drug administered to mice of group III revealed a noteworthy (^###^ *p* < 0.001) rise in GR (Table 2).

#### 2.3.5. Reduced Glutathione Levels (GSH)

We observed a substantial (*** *p* < 0.001) deterioration in GSH of group II mice in comparison to group I. A substantial (^#^ *p* < 0.05) rise was seen in mice of group IV that had received zingerone (25 mg/kg) while a healthy rise in (^###^ *p* < 0.001) levels of GSH was seen in mice administered zingerone (50 mg/kg) compared to levels in the toxic group that had received LiCl and pilocarpine only (group II). Sodium valproate, the standard antiepileptic drug administered to mice of group III demonstrated a significant (*** *p* < 0.001) increase in GSH in comparison to group II mice (Table 2).

#### 2.3.6. Effect of Zingerone and Sodium Valproate on Acetylcholine Esterase (AChE), Myeloperoxidase (MPO), Reactive Oxygen Species (ROS), and Nitrite (NO) Levels in LiCl-and-pilocarpine-induced Epilepsy

**Figure 2:**
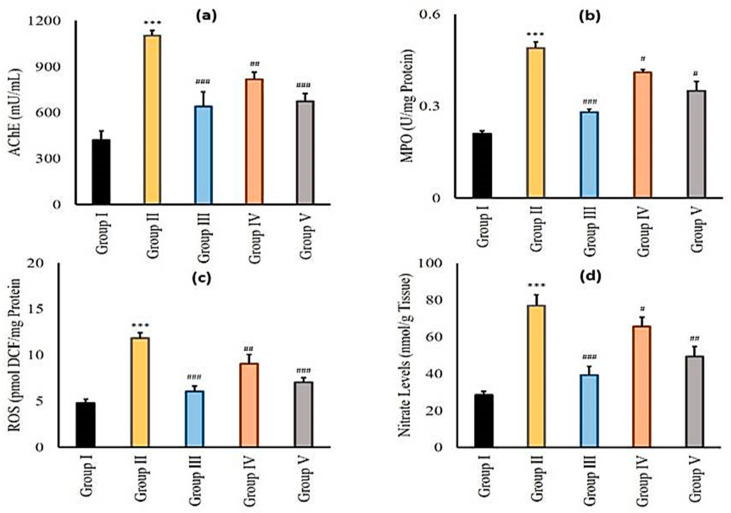
Panel of graphs represents effects of zingerone on AChE (**a**), MPO (**b**), ROS (**c**), and NO (**d**) levels on LiCl-and-pilocarpine-induced SE. Values are significantly different in LiCl and pilocarpine group (*** *p* < 0.001) as compared to control group. Results that we got are significantly different from LiCl and pilocarpine group as the main comparison is with LiCl and pilocarpine group only (^#^ *p* < 0.05, ^##^ *p* < 0.01 and ^###^ *p* < 0.001). Group I: Normal saline (10 mL/kg b.w.), Group II: LiCl (3 mEq/kg b.w.) + pilocarpine (30 mg/kg b.w.), Group III: Sodium Valproate (300 mg/kg b.w.) + LiCl (3 mEq/kg b.w.) + pilocarpine (30 mg/kg b.w.), Group IV: zingerone (25 mg/kg b.w.) + LiCl (3 mEq/kg b.w.) + pilocarpine (30 mg/kg b.w.), Group V: zingerone (50 mg/kg b.w.) + LiCl (3 mEq/kg b.w.) + pilocarpine (30 mg/kg b.w.)

AChE, MPO, ROS, and NO showed significant elevation (*** *p* < 0.001) in LiCl and pilocarpine administered in group II when compared with negative control. Sodium valproate, the standard antiepileptic drug administered to mice of group III exhibited significant decrease (^###^ *p* < 0.001) in AChE, MPO, ROS, and NO in comparison with group II (Figure 2a–d). Zingerone (25 mg/kg) in group IV showed (^#^ *p* < 0.05, ^##^ *p* < 0.01, ^###^ *p* < 0.001) significant fall in AChE, MPO, ROS, and NO activities. In group V, zingerone administration (50 mg/kg b.w.) revealed significant (^#^ *p* < 0.05, ^##^ *p* < 0.01, ^###^ *p* < 0.001) diminution in AChE, MPO, ROS, and NO activities in comparison with group II (Figure 2a–d).

#### 2.3.7. Effect of Zingerone and Sodium Valproate on Inflammatory Mediators (NFκB, TNF-α, IL-6 and IL-1β) in LiCl-and-pilocarpine-induced Epilepsy

Administration of LiCl and pilocarpine boosted (^***^ *p* < 0.001) all the inflammatory markers analyzed in our study (viz NFκ-B, TNF-α, IL-6 and IL-1 β). Pre-treatment of standard antiepileptic drug sodium valproate to mice of group III exhibited substantial (^###^ *p* < 0.001) reduction in all inflammatory marker levels in comparison with group II (Table 3). Zingerone (25 mg/kg b.w.) in group IV lowered increased levels of inflammatory markers NFκ-B (^#^ *p* < 0.05), TNF-α (^##^ *p* < 0.01), IL-6 (^###^ *p* < 0.001) and IL-1β (^#^ *p* < 0.05). In group V, zingerone (50 mg/kg b.w.) treatment pointedly decreased inflammatory markers viz NFκ-B (^###^ *p* < 0.001), TNF-α (^###^ *p* < 0.001), IL-6 (^##^ *p* < 0.01) and IL-1β (^###^ *p* < 0.001) significantly (Table 3).

From the above results, we conclude that zingerone (50 mg/kg b.w.) demonstrates better protection in LiCl and pilocarpine administration. So, for further histopathological and immunohistochemistry experiments, we have selected only the higher dose of zingerone that is 50 mg/kg b.w.

### 2.4. Histopathological Analysis of Zingerone and Sodium Valproate Treatment in LiCl-and-pilocarpine-induced Epilepsy

**Figure 3:**
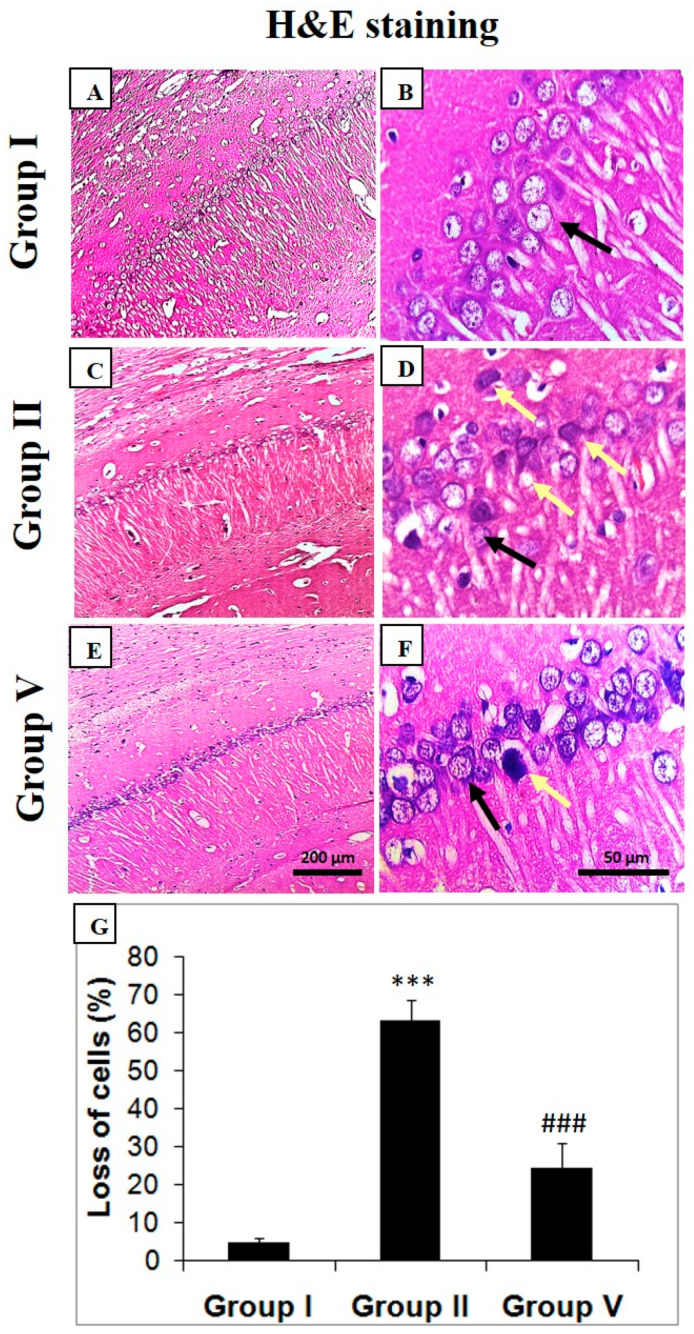
Zingerone effects on histomorphological features in LiCl-and-pilocarpine-induced SE. There is normal histology of group I (**A**,**B**) with intact and appropriately sized nuclei and neural cells. In group II (**C**,**D**) there is distortion of neurons along with neural condensation and necrosis, pyknotic nuclei in pilocarpine-administered rats as compared to negative control mice. Group V (**E**,**F**) treatment of zingerone (50 mg/kg) recovered the damage which was evident by retaining of normal histology and decrease in neural distortion and death. Values are expressed as mean ± SEM (*n* = 6). Photomicrographs of hippocampus depicting Hematoxylin and eosin staining analyses. Below photomicrographs is the panel which shows quantitative evaluation of neural loss. Significant difference was indicated by *** *p* < 0.001 when compared with group I and (^###^ *p* < 0.001) when compared with group II. Zingerone treatment significantly protected neural loss in group V (^###^ *p* < 0.001) when compared with group II (**G**). Group I: Normal saline (10 mL/kg b.w.), Group II: LiCl (3 mEq/kg b.w.) + pilocarpine (30 mg/kg b.w.), Group V: zingerone (50 mg/kg b.w.) + LiCl (3 mEq/kg b.w.) + pilocarpine (30 mg/kg b.w.).

Histological examination of hippocampal sections revealed normal morphology of neurons of group I animals. No deformity in CA1 sections of normal mice was observed and healthy neurons are marked by black arrow (Figure 3). Neural condensation, necrosis, and nuclear degeneration in the CA1 region were observed in the hippocampus of mice of group II animals that had received LiCl and pilocarpine only as indicated by yellow arrows (*** *p* < 0.001). Mild condensation and less degeneration of neurons were observed in the hippocampus of mice of group V that had been administered 50 mg/kg b.w. of zingerone in comparison with the positive control (^###^ *p* < 0.001) (Figure 3). 

### 2.5. Effect of Zingerone on Choline Acetyl Transferase (ChAT), Bcl-2, and Caspase Expression

**Figure 4:**
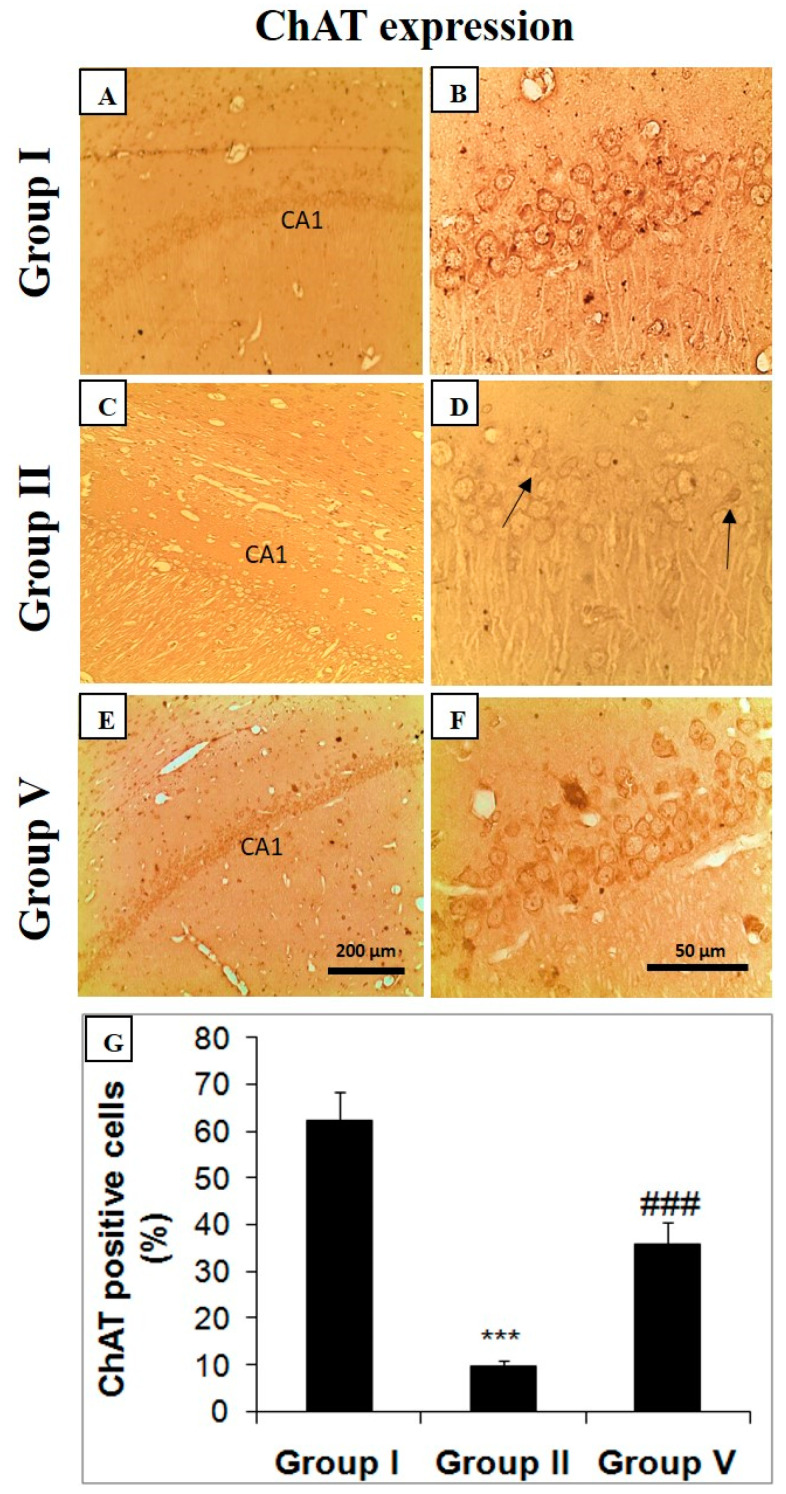
Effect of zingerone on choline acetyl transferase (ChAT) in LiCl-and-pilocarpine-induced SE. Photomicrographs of hippocampus depicting immunohistochemical analyses. Below photomicrographs is the panel which shows quantitative evaluation of ChAT. Values are expressed as mean ± SEM (*n* = 6). Significant differences were indicated by ^###^ *p* < 0.001 when compared with group II. Brain sections showing immunohistochemical results demonstrate specific immune positive staining of ChAT with brown color. The CA1 section of hippocampus in LiCl and pilocarpine-administered group-II (**C**,**D**) has decreased immunopositive staining of ChAT as quantified by brown color in comparison to negative control (**A**,**B**). But treatment of zingerone (50 mg/kg b.w.) in group V (**E**,**F**) increased ChAT immune-staining compared to group II (### *p* < 0.001) (**G**). Group I: Normal saline (10 mL/kg b.w.), Group II: LiCl (3 mEq/kg b.w.) + pilocarpine (30 mg/kg b.w.), Group V: zingerone (50 mg/kg b.w.) + LiCl (3 mEq/kg b.w.) + pilocarpine (30 mg/kg b.w.); *** *p* < 0.001.

**Figure 5:**
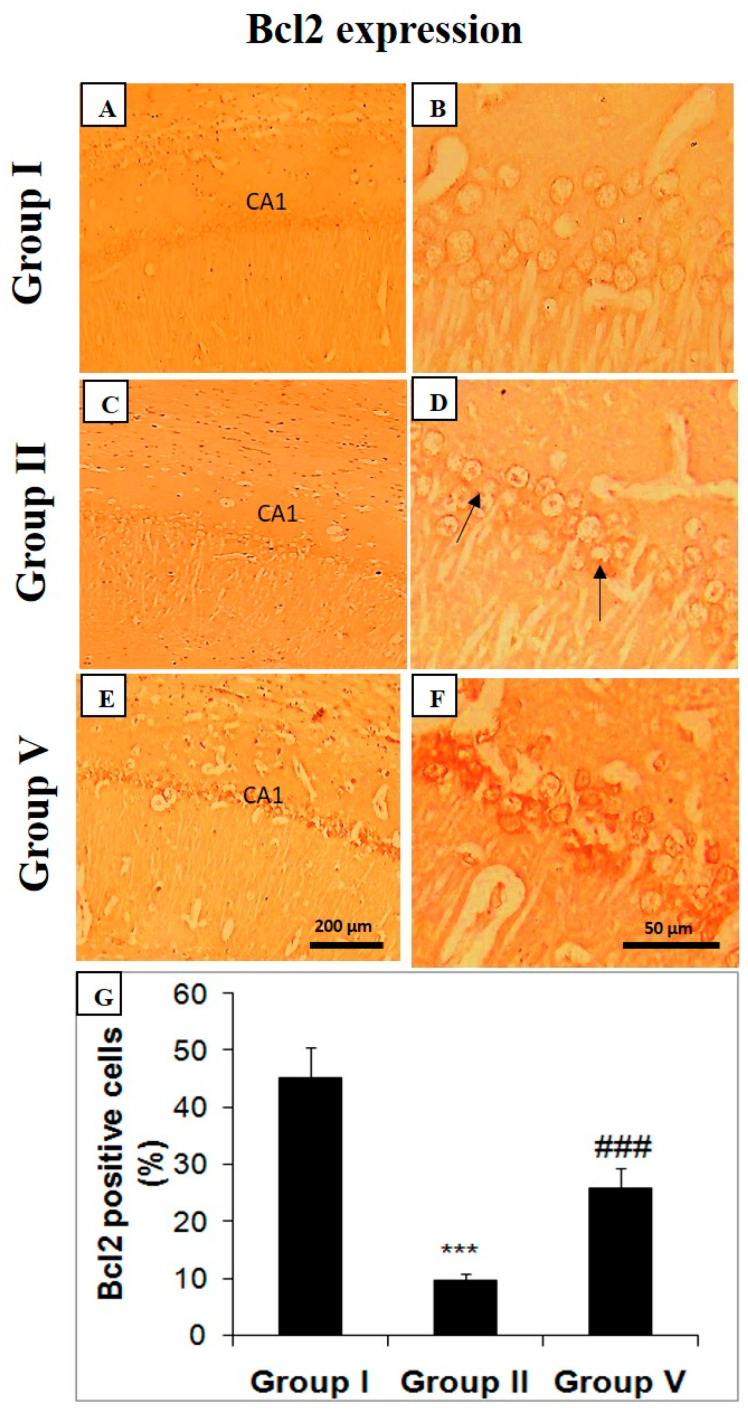
Zingerone upregulates Bcl-2 expression in LiCl-and-pilocarpine-induced SE. Photomicrographs of hippocampus depicting immunohistochemical analyses indicating specific immune positive staining of Bcl-2 with brown color. Below photomicrographs is the panel which show quantitative evaluation of Bcl-2. Values are expressed as mean ± SEM (*n* = 6). Significant differences were indicated by *** *p* < 0.001 when compared with group I and (### *p* < 0.001) when compared with group II. The CA1 section of hippocampus in LiCl and pilocarpine administered group-II (**C**,**D**) has decreased immuno-positive staining of Bcl-2 as specified by brown color in comparison to negative control (group I) (**A**,**B**). However, treatment with zingerone (50 mg/kg b.w.) in group V (**E**,**F**) enhanced immune positive staining of Bcl-2 in comparison to positive control (group II) (### *p* < 0.001) (**G**). Group I: Normal saline (10 mL/kg b.w.), Group II: LiCl (3 mEq/kg b.w.) + pilocarpine (30 mg/kg b.w.), Group V: zingerone (50 mg/kg b.w.) + LiCl (3 mEq/kg b.w.) + pilocarpine (30 mg/kg b.w.).

The deficiency in cholinergic activity is reported by the decrease in the ChAT expression in the hippocampal region of the brain in LiCl and pilocarpine-administered mice (group II). The black arrows represent the neural loss and decreased ChAT activity (Figure 4). Zingerone at the 50 mg/kg treatment enhanced the expression of ChAT activity in the hippocampus of mice in comparison with the positive control (group II) (Figure 4). Bcl-2 expression was upregulated in the hippocampus of LiCl and pilocarpine-administered mice in comparison with the control group (Figure 5). Zingerone as 50 mg/kg successfully downregulated the Bcl-2 activities in group V treated with the higher dose of zingerone (Figure 5) as compared to the positive control (group II) demonstrating the survival of neurons. Hence, there was an increase in the survival of neurons.

**Figure 6:**
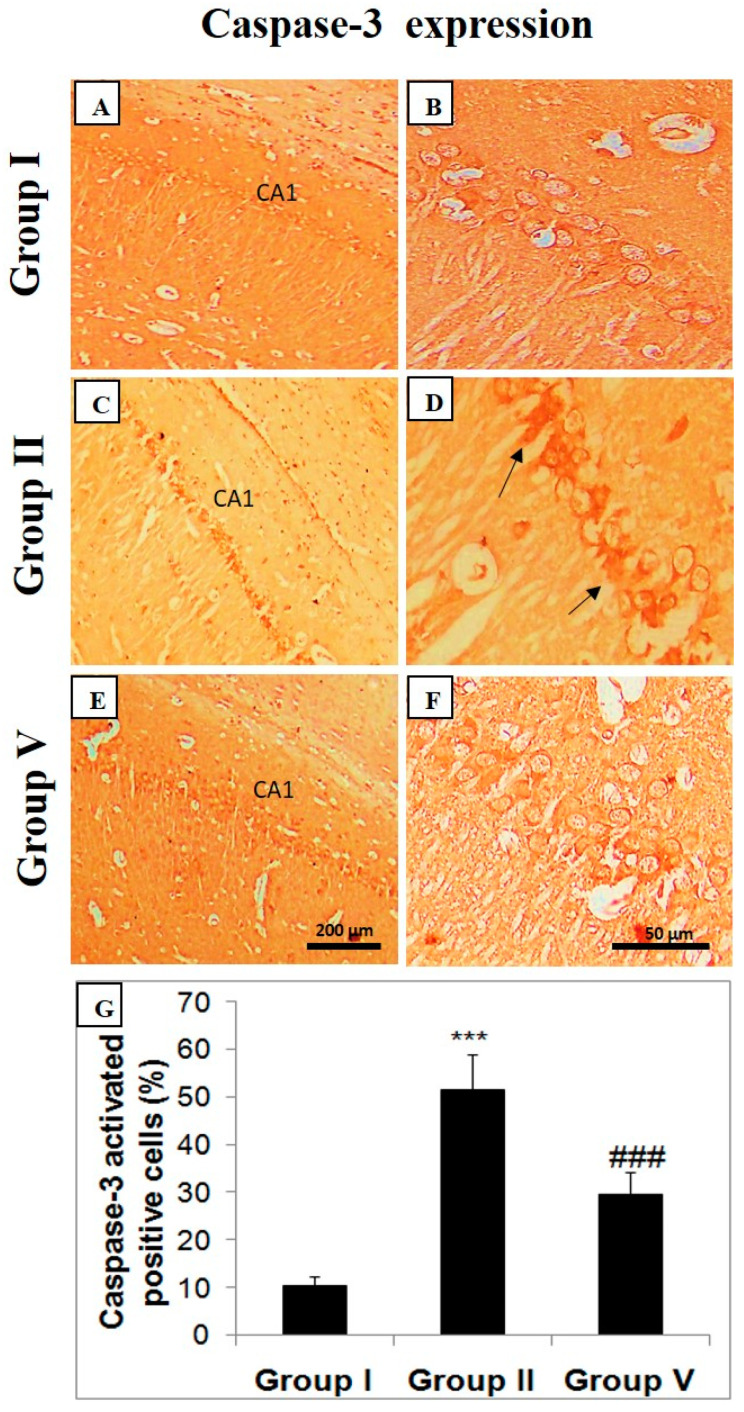
Zingerone downregulates caspase-3 expression in LiCl-and-pilocarpine-induced SE. Photomicrographs of hippocampus depicting immunohistochemical analyses. Below photomicrographs is the panel which shows quantitative evaluation of activated caspase-3 indicating specific immune positive staining of activated caspase-3 with brown color. Values are expressed as mean ± SEM (*n* = 6). Significant differences were indicated by *** *p* < 0.001 when compared with group I and (^###^ *p* < 0.001) when compared with group II. The CA1 section of hippocampus in LiCl and pilocarpine administered group-II (**C**,**D**) has enhanced caspase-3 immuno-positive staining as stipulated by brown color in comparison to negative control group (group I) (**A**,**B**). But zingerone treatment (50 mg/kg b.w.) in group V (**E**,**F**) decreased activated caspase-3 in comparison to positive control (group I) (^###^ *p* < 0.001) (**G**). Group I: Normal saline (10 mL/kg b.w.), Group II: LiCl (3 mEq/kg b.w.) + pilocarpine (30 mg/kg b.w.), Group V: zingerone (50 mg/kg b.w.) + LiCl (3 mEq/kg b.w.) + pilocarpine (30 mg/kg b.w.).

Negligible activated caspase-3-positive neurons were visible in the control group (group I) (Figure 6). In group II, with the treatment of LiCl and pilocarpine in mice, activated caspase-3 expression is upregulated in the hippocampal region of the brain (Figure 6). Black arrows show degenerated neurons with high expression of activated caspase-3. Zingerone at the 50 mg/kg attenuated caspase-3 expression in the group treated with a higher dose of zingerone as juxtaposed to a positive control (group II) indicating survival of neurons (Figure 6).

## 3. Discussion

Initial pathology of SE involves unwarranted ROS production, strong neuroinflammatory retorts, discerning neural deterioration, and interruption of BBB, following consequent impairment in cognition [23]. The purpose of SE treatment is to terminate seizures as quickly as possible with either first-line or second-line drug therapy such as anti-depressants and anti-epileptic drugs which include phenobarbital or valproic acid, respectively. Apparently, these drugs terminate seizures immediately. However, they do not efficiently recover the pathophysiological variations which occur following epileptic seizure episodes leading to neural injury and cognitive deficits [24]. Consequently, new treatment approaches are highly needed to mitigate the pathophysiological damage in the brain after SE. We hypothesized that zingerone may provide beneficial outcomes in SE centered on previous studies [25]. 

Seizure intensity is assessed usually in seizure models of animals to do behavioral analysis. One of the most frequently used seizure intensity measurements in epilepsy is done by Racine’s scale. This scale has become efficient over the years to better define the development of seizures initiated by various chemicals [26]. Zingerone treatment demonstrated a reduction in the score of seizures and a boost in latency of seizures. The results were more significant and comparable with sodium valproate when higher doses of zingerone were administered. The function of sodium valproate is to enhance GABAergic activity besides diminishing excitatory neurotransmission [27]. The effect of zingerone in altering epileptogenesis could be attributed to its role in decreasing stimulatory neural transmission, elevating GABAergic activity, and perturbing ion-channel activity, as reported previously [28,29,30,31,32]. In the case of epileptogenesis, there are reports of decreased GABAergic activity of neurons leading to seizures [33]. Zingerone has been previously reported in the seizure model of mice to enhance the GABAergic activity by an increase in the Ca^++^ influx through ionophores of presynaptic 5HT3 receptors causing an anticonvulsant effect [34]. Similarly, the ion channels are also reported to be dysfunctional in epilepsy-inducing seizures [35]. Zingerone has prevented the ion channel disruptions, as previously reported by Hosseini and Mirazi [33]. However, the exact mentioned mechanism by which zingerone acts in the present model of epilepsy is not clear and needs further study.

One of the clinical manifestations in SE-induced brain damage is cognitive impairment. Long-term seizures which occur repeatedly over a long period of time in SE inescapably have an impact on patients and affect their quality of life. Unfortunately, AEDs do not alleviate memory deficit caused by seizures and their long-term usage elevates oxidative stress and prompts cognitive imbalance. Moreover, epilepsy itself contributes to oxidative stress, which is further heightened by AEDs, indicating that antioxidants, anti-inflammatory, and anti-apoptotic agents may have the potential to impede seizures and the pathophysiology associated with SE deciphering antiepileptic therapy [36]. The Morris water maze (MWM) is the interpretation of memory and learning. LiCl-and-pilocarpine-induced SE lowers the behavioral pattern of learning as evidenced by higher values of escape latency in the positive control group, which, however, was counteracted by zingerone in groups IV and V, suggesting the protective role of zingerone in the improvement of neural damage. Results of the MWM reveal that positive control animals either stayed for a smaller amount of time in the target quadrant or took a longer amount of time to grasp the escape stand or entirely could not grasp the stand, indicating poor cognition and visual-spatial memory, as reported previously [7,37], when compared with control animals. Moreover, the explicit basis of poor cognition following SE is not known. Nevertheless, imbalances in neurochemical signaling and progressive structural damage to neurons induced by recurrent transitory seizures in SE may be one of the reasons for neurobehavioral manifestations. Our results are further validated by previous findings.

One of the hallmarks and fundamental cytotoxic mechanism of recurrent epileptic seizure is the induction and activation of oxidative stress which further exacerbate neural toxicity, dysfunction, and death. It is well evidenced from augmented oxidized proteins and lipid levels found clinically in epilepsy patients. However, oxidation of biomolecules that contribute to excitatory neural damage and collapse was also found in epileptic brain tissue surgically resected, indicating that oxidative stress is an important phenomenon in SE [38]. Pilocarpine results in surging ROS, NO content, and lipid peroxidation formation, as reported previously. It decreases antioxidants like nitric oxide synthases (NOS), CAT, SOD, GR, GSH amounts in different areas of the brain, which include the hippocampus, cortex, or other areas or entire brain. We also obtained similar results in the present study as ones reported previously. However, antioxidant therapy is found to exhibit beneficial effects on various epilepsy models by ameliorating oxidative stress in the brain [39]. Similar results were obtained in our current study. The brain consumes the largest quantity of oxygen in comparison to the rest of the areas of the body which makes it prone to redox imbalance. It also comprises a large amount of polyunsaturated fatty acids, therefore, it is predisposed to peroxidative lipids [8]. SOD, CAT, GPx, GR are endogenous antioxidant machinery enzymes implicated in the cleansing of ROS where superoxide anion radicals are dismutated by SOD, H_2_O_2_ is decomposed by CAT decreasing oxidative stress, effectively protecting itself from redox damage. The hydroxyl ion (OH^0^), which is the most damaging free radical, attacks molecules by hydrogen abstraction, subsequent repeated attacks trigger injury to genetic material, proteins, lipids, and cellular membranes of mitochondria and nucleus. H_2_O_2_ indeed is a non-free radical; nevertheless, it is exceptionally detrimental and has the ability to traverse lipid layers, reacting with transition metals. Besides, it can stimulate chromosomal modifications and oxidize sulfhydryl compounds (in the absence of catalase). In the present study, we show increased H_2_O_2_ formation in pilocarpine-induced group in comparison with negative control, as stated before [40]. However, treatment with zingerone scavenged H_2_O_2_ production, thereby, a decrease in content and similar results were obtained in the sodium valproate group. GPx shields cells against oxidative damage and meanwhile involves GSH as its cofactor. GSH is a highly recognized non-enzymic antioxidant that defends against peroxidative damage of cell membranes endogenously [41]. In the present study, we found a decrease in SOD, CAT, GR, and GSH in LiCl-and-pilocarpine-induced group as reported previously [42]. Administration of zingerone notably in high doses or sodium valproate diminishes oxidative damage with the restoration of the antioxidant armory. These results are in agreement with earlier reports [24,37,43,44].

The oxidative stress and generation of free radicals in epilepsy has been established. Pearson-Smith et al., Diniz et al., and Clayton et al. studied oxidative stress in epilepsy models [38,39,40], and there has been increasing evidence supporting the use of several natural compounds in combating the ROS generated by unstable radicals as described by Rehman et al., Shakeel et al., Martinc et al., Mani et al., Carmona-Aparicio et al. in neurological and other disease models [11,36,41,42,43]. However, our study is novel in establishing the neuroprotective and antioxidant role of zingerone in the SE model of mice targeting inflammation, neural cell death, and redox imbalance.

The role of the cholinergic system is well documented in pilocarpine-induced SE. AChE is critically involved in cholinergic neural communication and breakdown of acetylcholine to dismiss the propagation of neural signals. When acetylcholine gets accumulated, there is an activation of muscarinic and nicotinic receptors excessively. Therefore, an elevated cholinergic signal in the brain is associated with SE [45]. The increase in the activity of AChE in epilepsy is well documented. Studies have confirmed the upregulation of AChE allied with elevated immune response, facilitating the epileptogenic process [46]. The anti-inflammatory role of zingerone in the present work is presumed to be associated with the downregulation of AChE, elevating the Ach levels which in turn inhibit the innate immune response a1nd proinflammatory cytokines. We hypothesize the role of zingerone in improving cholinergic dysfunction, possibly by downregulation of AChE; this needs further research to uphold.

Inflammation in the neurons plays a vital role in epileptogenesis, which has been well evidenced by clinical signs and neuropathology events in epilepsy in the last decade. Pro-inflammatory cytokines have been shown remarkably to be the most upregulated molecules during epileptogenesis. Neural inflammatory molecules elevate neural excitability via augmenting synaptic transmission by mediating glutamate receptors leading to downregulation of seizure threshold [47]. Henceforward, one of the objectives of the current work was to elucidate the functional importance of NF-κB and inflammatory mediators in LiCl-and-pilocarpine-induced epilepsy. NF-κB, a redox-sensitive molecule that is directly proportional to pro-inflammatory mediators’ expression in neural excitability and gliosis in the hippocampus. Therefore, it becomes a practically medicinal target in the management of epilepsy [48]. In the current work, we examined NF-κB in the hippocampus region. Our results decipher that NF-κB is substantially elevated in LiCl-and-pilocarpine-induced epilepsy in comparison with the negative control. Though, prophylactic treatment with zingerone downregulated NF-κB in the hippocampi regions of mice as reported previously [9]. The elevated inflammatory marker levels like TNF-α, IL-1β, and IL-6 in toxic control group are attributed to activation of the innate immune response. Any ischemic, epileptic, or excitotoxic damage causes initiation of proinflammatory cytokine storm including TNF-α, IL-6, IL-1β as reported previously [47,49]. Our study confirmed it further that there was an increase in cytokine levels in the LiCl and pilocarpine group, indicative of inflammatory reactions leading to seizures. However, treatment with zingerone alleviated cytokine storm as reported previously by Rehman et al. and Wali et al. [50,51] and therefore regulating neuroinflammation and, hence, controlling seizures. 

Neuroinflammation induced by epileptic seizures results in neural death in several brain regions, particularly the hippocampus. If inflammation persists excessively it results in cellular damage and neurotoxicity, as reported in the literature. However, the limitation of the present study is that we have not studied specific marker proteins of microglia and glial cells, which makes it hard to be certain about a particular cell’s involvement. It has been reported that microglial activation facilitates the release of cytokines and chemokines like NF-κB, TNF-α, and IL-1β which is found in the current study, as reported previously [52]. 

Instigation of inflammatory pathways like the NO pathway in various experimental and clinical studies has been found to have a critical position in the pathology of epileptic seizures and neural lesions. NO is a neurotransmitter in the brain found in the gaseous state and NOS regulates the biosynthesis of NO. NO is a free radical which has low reactivity with most of the biomolecules. Nonetheless, it may react with other free radicals, generating peroxynitrite. The latter is a potent inducer of cell death [53,54]. Our results reveal that NO content in the hippocampus is augmented following seizures, representative of epileptic seizures. However, zingerone treatment reduces NO content, alleviating inflammation and toxicity of NO on brain tissue, as reported previously by Mir et al. [55]. 

Myeloperoxidase is an inflammatory marker found in leucocytes and is the only peroxidase that uses H_2_O_2_ to oxidize halides forming hypohalous acids. So, it involves the production of RNS and ROS. It also inhibits inhibitors of matrix metalloproteinases, leading to leakage in BBB, which is associated with the advancement and initiation of seizures. We show an increase in MPO activity in positive control animals, as reported previously [56]. However, treatment with zingerone alleviated MPO content, indicating the anti-inflammatory role of zingerone, as reported [57].

Apoptosis related to redox signaling is associated with epilepsy. Induction of apoptotic signaling pathway is reported to worsen seizure-induced brain damage resulting in extended epileptic seizures. In reaction to seizure-stimulated brain injury, permeabilization of mitochondria happens and proapoptotic proteins are secreted from mitochondria, leading to downstream executioner caspase (caspase-3) activation causing brain lesions [58]. Evidence suggests that there is cross-talk between oxidative stress and mitochondrial dysfunction followed by apoptosis in seizure-induced neural damage, making both changes in mitochondria and oxidative stress elements for epileptogenesis [59]. Furthermore, mitochondria are vulnerable to oxidant damage as they are predominant sites of ROS generation. Current data suggest that targeting oxidative stress, inflammation, and mitochondrial disruption may be a new approach for precluding neural death and inhibiting epilepsy, and controlling seizures [60,61]. The outcomes of zingerone on neural lesions following epileptic seizures by LiCl and pilocarpine administration, and on mitochondrial-apoptosis-pathway-related regulator Bcl-2 and caspase-3 were studied, which is an imperative procedure of neuroprotective potential of zingerone to lessen cell death and endorse survival as reported [19,20]. The results indicate that zingerone intervened and limited brain damage, increased Bcl-2 protein expression in hippocampal neurons, downregulated apoptotic protein activated caspase-3 expression, suggesting that zingerone regulates the expressions of these mitochondrial intrinsic apoptotic-pathway-associated molecules to diminish lesions and death of hippocampal cells and play a beneficial role in protection of brain [19,20].

Histology shows neural thickness was considerably diminished in the positive control group in the CA1 region of the hippocampus which may be attributed to the unnecessary increase of intracellular Ca^2+^ levels due to overtly activated glutamate receptors, as reported earlier [62]. However, treatment with zingerone in group V markedly increased neural density, indicating growth and neural survival. Apoptotic cell death, necrosis, and pyknotic nuclei are seen in positive control in comparison to the negative control. Additionally, to validate the hippocampal lesions in the brain, we also explored intrinsic-pathway-apoptosis-related factors like Bcl-2 and caspase-3 which showed that zingerone treatment augmented Bcl-2 and alleviated caspase-3 expression in group V [63].

## 4. Materials and Methods

### 4.1. Chemicals

Zingerone, lithium chloride, and other chemicals used in the study were obtained from Sigma Aldrich (St. Louis, MO, USA) and were having the largest purity proportion. Pilocarpine (3*S*,4*R*)-3-Ethyl-4-((1-methyl-1H-imidazol-5-yl)methyl)dihydrofuran-2(3H)-one was purchased from HiMedia and sodium valproate (sodium 2-propylpentanoate) from Unicure Remedies Pvt. Ltd (Gorwa, Baroda, India).

### 4.2. Animal Study

Male swiss albino mice weighing 25–35 g, of 4–6 weeks old, were housed in the institutional animal facility, having easy access to food and drinking water with a relative humidity of 45–55% in a temperature of 23–25 °C of 12 h dark/12 h light period. Institutional Animal Care and Ethics Committee (No: RAKMHSU-REC-08-2019-F-P) permitted all the experimental procedures described in the manuscript. Animals were randomly divided into five groups of 14 mice in each group.

### 4.3. Preparation of Drugs and Treatment Protocol

**Figure 7:**
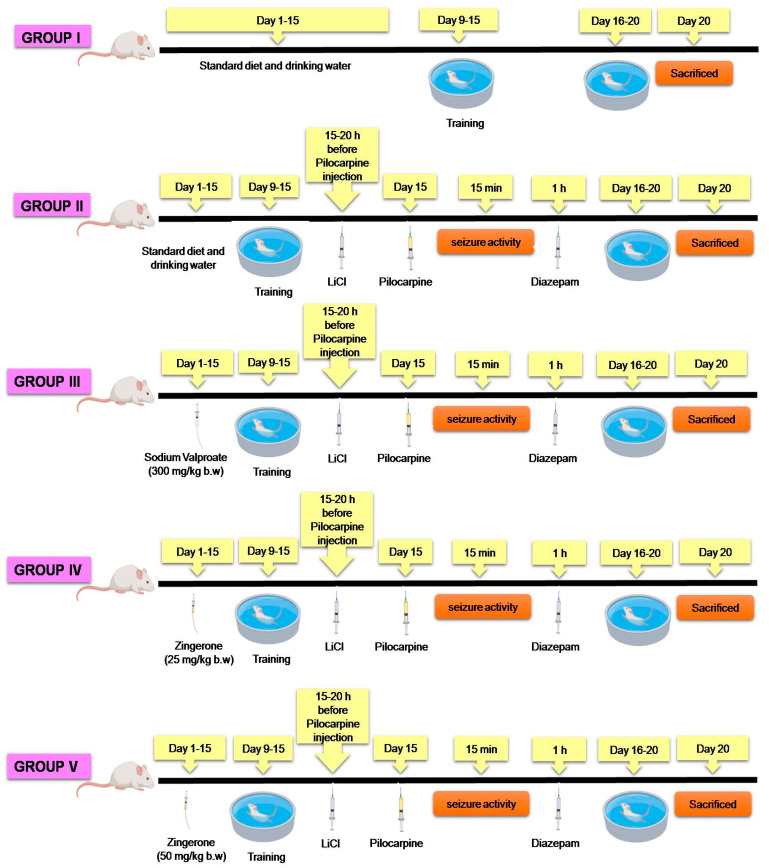
A schematic of status epilepticus induced by pilocarpine treatment protocol.

Pilocarpine was used to induce epilepsy and was freshly prepared in water and given intraperitoneally (i.p.) at the dose of 30 mg/kg body weight to mice. Lithium chloride (3 mEq/kg, i.p.) was administered 15–20 h prior to pilocarpine injection. Lithium potentiates pilocarpine effect by activating T-lymphocytes and mononuclear cells indirectly, resulting in higher serum IL-1β levels, thus varying the BBB permeability and augmenting pilocarpine uptake. Researchers have also found pre-treatment with LiCl to increase acetylcholine release leading to more acetylcholine crossing the synaptic cleft and reaching the postsynaptic membrane, activating muscarinic receptors and decreasing time to SE onset. The LiCl pilocarpine model has been shown to generate more consistent and prolonged seizures with reproducible results with a low mortality rate, thus making it a promising model for studying SE [64]. Sodium valproate was used as a standard antiepileptic drug, which was prepared in a similar way as pilocarpine and was given at the same dose to one of the groups to compare the results with our natural compound group. Zingerone was freshly prepared in normal saline and given as 25 and 50 mg/kg body weight based on previous reports [56,65,66,67] For the treatment schedule, refer to Figure 7. Control animals were treated with an equivalent volume of water (10 mL/kg b.w.) for 15 days preceding pilocarpine [68].

#### Experimental Regimen

Before the start of the experiment, all animals were trained in the Morris water maze. The animals were randomly selected into five groups of fourteen animals each. 

Group I: Negative control. Mice were provided with the standard diet with free access to oral drinking water.

Group II: Positive control/pilocarpine group. In this, mice were provided with a standard diet and drinking water. Pilocarpine was injected 30 mg/kg b.w i.p. on the 15th day. LiCl (3 mEq/kg b.w.) was given intraperitoneally 15–20 h before pilocarpine injection.

Group III: Standard drug group. In this group, mice were provided with a standard diet and drinking water. LiCl (3 mEq/kg b.w.) was given intraperitoneally 15–20 h before pilocarpine injection. Pilocarpine (30 mg/kg b.w.) i.p. was given on day 15, and Sodium Valproate (300 mg/kg b.w.), which is a standard anti-epileptic drug, was given prophylactically orally for 15 days prior to pilocarpine injection.

Group IV: (Prophylactic Group I). Mice were given a standard diet and drinking water. LiCl (3 mEq/kg b.w.) was given intraperitoneally 15–20 h before pilocarpine injection. Pilocarpine (30 mg/kg b.w.) i.p. was given on day 15, and zingeron as 25 mg/kg b.w. was administered for 15 days orally prior to pilocarpine injection from the start of the study.

Group V: (Prophylactic Group II). Mice were given a standard diet and drinking water. LiCl (3 mEq/kg b.w.) was given intraperitoneally 15–20 h before pilocarpine injection. Pilocarpine (30 mg/kg b.w.) i.p., given on day 15 and zingerone as 50 mg/kg b.w, administered for 15 days orally prior to pilocarpine injection from the start of the study. 

After administering pilocarpine, the alterations in seizure activity in different groups were recorded for a period of 1 h as per the following Racine classification [68]. SE was terminated by diazepam (10 mg/kg, i.p.) Eight animals from each group were used for biochemical estimations, and six animals were used for histopathology and immunohistochemistry studies. Furthermore, for histology and immunohistochemistry studies, we proceeded with only three groups on the basis of the best results that we got from our biochemical and behavioral studies, and we found the higher dose of zingerone to be the best for mitigating the process of SE.

Prophylactic activity of zingerone was measured for modifying SE by checking cognitive impairment by MWM, ROS measurement, antioxidant armory, seizure severity, latency to onset of seizures, NFκ-B, inflammatory mediators like TNF-α, IL-1β, and apoptosis pathway proteins; histological alterations were also studied.

### 4.4. Morris Water Maze (MWM)

The already trained mice were assessed for spatial learning and memory abilities. The test was performed for 5 days. MWM (TSE Systems, Chesterfield, MO, USA) is comprised of a round pool with water (25 ± 2 °C) in which mice are trained to swim to a concealed stand and flee from water, as described previously [69]. Before the start of experiments, animals were trained to find the hidden platform by giving four trials each day for each animal for five consecutive days. The platform was fixed at one place permanently during the complete procedure. When animals climbed at the platform, it remained there for 15 s prior to the beginning of the subsequent trial. If in the maximum allowed time of 60 s, mice failed to grasp the escape stand, it was quietly put on the platform and allowed to stay there for 15 s prior to its removal from the pool. Escape latency is the total amount of time required by mice here to grasp the concealed stand, which was evaluated in seconds. 

Spatial probe test: The probe test was performed by eliminating the hidden platform from the pool, and mice were permitted for 60 s to freely swim in the pool. The amount of time devoted by the mice in the target quadrant was estimated, and this was the measure of the intensity of memory reinforcement following learning. 

### 4.5. Tissue Homogenization

Once behavioral studies were done, animals were sacrificed, the hippocampus blotted dry, weighed (5% *w*/*v*), and homogenized in 0.01 mM phosphate-buffered saline having pH 7.4 with 10 μL/mL protease inhibitor cocktail in Potter-Elvehjem homogenizer (Thomas Scientific, Swedesboro, NJ, USA) and centrifuged (TSE Systems, Chesterfield, MO, USA) at 825× *g* for 5 min at 4 °C to separate debris. The supernatant-1 (S1) was used for the assay of lipid peroxidation, myeloperoxidase, and AChE activity, and the rest was again centrifuged in REMI cooling centrifuge to acquire post mitochondrial fluid (PMF/PMS) of the hippocampus at 10,500× *g* for 15 min at 4 °C, which was used for the estimation of the antioxidant armory, NO, and inflammatory markers. The absorbance was calculated by UV-1601 (Shimadzu, Japan) for the antioxidant armory and for inflammatory markers by ELISA Plate Reader (benchmark plus, Biorad, Hercules, CA, USA) [20].

#### 4.5.1. Assessment of Lipid Peroxidation (LPO)

LPO was calculated by Wright et al. protocol [70]. The reaction mixture contained 200 μL S1, 200 μL ascorbic acid of 100 mM, 580 μL phosphate buffer of 0.1 M, pH 7.4, and 20 μL ferric chloride of 100 mM, making 1 mL as total volume. This reaction was incubated at 37 °C kept in a shaking water bath for 1 h. By adding 10% of 1 mL, the trichloroacetic acid reaction was stopped, followed by the addition of 0.67% of 10 mL thiobarbituric acid (TBA), and all the reactions in tubes/vials were placed in a boiling water bath for a period of 20 min. The tubes/vials of the above-mentioned reaction mixture were transferred to an ice bath and then centrifuged at 2500× *g* for 10 min, and the absorbance of each sample was taken. 

#### 4.5.2. Estimation of Superoxide Dismutase Activity (SOD)

SOD was assessed by Marklund and Marklund protocol [71]. The total reaction mixture consisted of 100 μL PMS with 2875 μL Tris–HCl buffer of 50 mM of pH 8.5, pyrogallol with 24 mM in 10 mM-HCl, making reaction mixture in total of 3 mL, and the absorbance was taken.

#### 4.5.3. Estimation of Catalase Activity (CAT)

CAT was calculated by Claiborne protocol [72]. The reaction mixture consists of 50 μL of 5% PMS with 1950 μL phosphate buffer of 0.1 M concentration of pH 7.4, 1000 μL H_2_O_2_ of 0.10 mM making reaction mixture of 3mL in total and then absorbance is taken for each sample in each group. 

#### 4.5.4. Estimation of Glutathione Reductase [GR] Activity

GR was done as calculated by Rashid et al. [73]. The reaction mixture consists of 825 μL phosphate buffer of 0.1 M with pH 7.6, 50 μL EDTA of 0.5 mM, 25 μL oxidized glutathione of 1.0 mM, 50 μL NADPH of 0.1 mM and 25 μL of 5% PMS in a total volume of 1.0 mL.

#### 4.5.5. Estimation of Reduced Glutathione (GSH)

GSH was calculated as done by Rashid et al. [73]. PMS was mixed with 4.0% sulfosalisylic acid in a 1:1 ratio (*v*/*v*). The reaction mixture was incubated for 60 min at 4 °C for and then centrifuged at 1200× *g* for 15 min at 4 °C. The reaction mixture consisted of 400 μL filtered aliquot, 2200 μL phosphate buffer of 0.1 M with physiological pH and 400 μL DTNB of 10 mM, making up 3 mL as total and then absorbance is taken for each sample in each group.

#### 4.5.6. Assay for Activity of AChE

AChE was estimated as defined by Ellman et al. [74]. Acetylcholine (ATC), is an artificial substrate which is broken down in the presence of AChE to release thiocholine. Thionitrobenzoic acid is formed by reaction of thiocholine with DTNB. The reaction mixture contained 2.6 mL of 0.1 M sodium phosphate buffer having pH 7.4, 100 μL of 10 mM DTNB, 20 μL of ATC, and 400 μL S1 making 3.12 mL volume in total. Absorbance is taken for each sample in each group.

#### 4.5.7. Assay for Myeloperoxidase Activity

The neutrophil quantification is measured as a level of myloperoxidase (MPO) activity and was carried using the Bradley et al. method [75]. Activity was measured by mixing 0.1 mL of the supernatant with 2.9 mL of 50 mM phosphate buffer having pH 6.0, containing 0.167 mg/mL 0-dianisidine dihydrochloride and 0.0005% hydrogen peroxide. Absorbance is taken for each sample in each group.

#### 4.5.8. Reactive Oxygen Species (ROS) Assessment

ROS measurement was observed by the oxidation of 2′7′-dichlorodihydrofluorescein diacetate (DCF-DA) to 2′7′-dichloro-fluorescein (DCFH2) as described by Chan-Min Liua et al. [76].

#### 4.5.9. Assay for Nitric Oxide (NO)

Equal amounts of supernatants from various groups and Griess reagent (0.1% *N*-(1-naphthyl) ethylenediamine dihydrochloride, 1% sulfanilamide, and 2.5% H_3_PO_4_) were mixed together. Total nitrites were spectrophotometrically measured at 540 nm after incubation of 10 min at room temperature in the dark. The nitrite concentration was calculated by NaNO standard curve [77].

#### 4.5.10. Estimation of Nuclear Factor kappa B (NFκ-B), Tumor Necrosis Factor alpha (TNF-α), Interleukin 6 (IL-6) and Interleukin 1 beta (IL-1β)

Commercially available kit (eBioscience, Inc. San Diego, CA, USA) was used to assess NFκ-B, TNF-α, IL-6, IL-1β. Tests were done on an ELISA plate reader (Benchmark Plus, Biorad, Hercules, CA, USA) as per the directions of the company supplying the kit.

#### 4.5.11. Assay for Protein Measurement

The protein was measured by Lowry et al. [78] with BSA reagent as a standard.

### 4.6. Histopathology and Counting of Neuronal Loss

After the experiment, the mice were anesthetized and perfused transcardially with chilled PBS (10 mM, pH 7.4) and then 4% paraformaldehyde. The fixed brains were removed and embedded in paraffin. The paraffin sections at the level of hippocampus (thickness 5 µm) were dewaxed and rehydrated by using different gradient of alcohol for hematoxylin-eosin (H&E) staining. The morphology of the pyramidal neurons in CA1 region of hippocampus was examined under light microscopy (Olympus BX50, Tokyo, Japan) [20].

Neuronal loss was estimated as described by Vaibhav et al. The pyknotic nuclei were defined as darkly stained punctate nuclei and those nuclei that were fragmented were counted as single nucleus. The cells with pyknotic nuclei were regarded as dead cells and not counted in viable cell counting under the microscopic field in each group. In each group, the number of live cells and dead cells were counted in five areas of CA1 region of hippocampus and the average was calculated. The percentage of dead cells to that of viable cells was regarded as neuronal loss. This process was repeated five times [20]. 

### 4.7. Immunohistochemical Staining of ChAT, Bcl-2, and Caspase-3

Immunohistochemistry was done to identify ChAT, Bcl-2, and caspase-3 protein expressions. Immunohistochemical staining protocol was done as explained by Rashid et al. [79]. Animals were given anesthesia and perfused transcardially for 20 min with 4% paraformaldehyde in normal saline (9%). Brains extracted and fixed in 4% paraformaldehyde in normal saline (9%) for another 24 h. Coronal section of the hippocampus was processed for immunohistochemical staining. We used the following antibodies as anti-mouse ChAT rabbit antibody (1:200) (Sigma-Aldrich, Taufkirchen, Germany), anti-mouse Bcl-2 polyclonal antibody (1:150) (Thermo Fisher Scientific, Waltham, MA, USA), anti-mouse caspase-3 polyclonal antibody (activated caspase-3) (1:200) (Thermo Fisher Scientific, Waltham, MA, USA) overnight incubation at 4 °C following the protocol by Rashid et al. Next day, the slides were washed three times in Tris buffers (pH-6.0) and were incubated with a biotinylated Goat Anti-Polyvalent Plus (Thermo Fisher Scientific, Waltham, MA, USA) for 30 min at room temperature. This step was followed by further washing in Tris buffer and incubation of slides at room temperature with Streptavidin Peroxidase Plus (Thermo Fisher Scientific, Waltham, MA, USA) that binds to the biotin present on the secondary antibody. After washing in Tris buffer, the immunostaining reaction product was developed using 3,30-diaminobenzidine (DAB Plus substrate, Thermo Fisher Scientific, Waltham, MA, USA). After immunoreactivity, slides were dipped in distilled water, counterstained with Harris hematoxylin and dried, and finally, the sections were mounted with DPX and covered with cover slips. The slides were ready to be observed under microscope. (Olympus BX50, Tokyo, Japan). The positive neural cells were counted at five different areas of CA1 region of hippocampus by microscope (magnification X 200) and the process was repeated five times. The cells expressing the protein were plotted as mean percentage of total cells counted [20,79,80].

### 4.8. Statistical Analysis

The data are displayed as the mean ± standard error of the mean (SEM) from all the groups individually. Analysis of variance (ANOVA) was utilized to determine the differences between groups followed by Tukey–Kramer multiple comparisons test. Criterion for statistical significance minimally is set at *p* < 0.05 for all comparisons unless stated otherwise.

## 5. Conclusions

Zingerone alleviated LiCl-and-pilocarpine-induced epilepsy pattern and showed neural survival and protection in the hippocampus of mice after SE by reducing redox imbalance, inhibition of expression of inflammatory mediators, and increasing survival of neurons by regulating apoptosis, which are closely associated with onset of seizures. Henceforth, zingerone deciphered promising therapeutic efficacy in SE mice, offering a novel beneficial window for mitigation of epilepsy in vivo.

## Figures and Tables

**Table 1:** Zingerone and sodium valproate effects on seizure severity and latency to onset of SE in LiCl-and-pilocarpine-induced epilepsy in mice.

Animals/Convulsion Score	Group I	Group II	Group III	Group IV	Group V
No. of Animals	14	14	14	14	14
Latency to First Convulsion (s)	0	564 ± 44	3473 ± 48 ***	864 ± 55 **	1194 ± 56 ***
Percentage Convulsion (%)	0	100	10	100	42.6

Results are representative of mean ± SE of fourteen mice per group. The results that we obtained are significantly different from the LiCl and pilocarpine group as the main comparison is with LiCl and pilocarpine group only (** *p* < 0.01 and *** *p* < 0.001). Group I: Normal saline (10 mL/kg b.w.), Group II: LiCl (3 mEq/kg b.w.) + pilocarpine (30 mg/kg b.w.), Group III: Sodium Valproate (300 mg/kg b.w.) + LiCl (3 mEq/kg b.w.) + pilocarpine (30 mg/kg b.w.), Group IV: zingerone (25 mg/kg b.w.) + LiCl (3 mEq/kg b.w.) + pilocarpine (30 mg/kg b.w.), Group V: zingerone (50 mg/kg b.w.) + LiCl (3 mEq/kg b.w.) + pilocarpine (30 mg/kg b.w.).

**Table 2:** Zingerone treatment effects on antioxidant enzymes and lipid peroxidation on LiCl-and-pilocarpine-induced SE in experimental mice.

Parameters	Group I	Group II	Group III	Group IV	Group V
LPO (n moles MDA formed/g tissue)	2.92 ± 0.03	6.09 ± 0.42 ***	3.32 ± 0.43 ^###^	4.80 ± 0.54 ^#^	3.43 ± 0.54 ^###^
SOD (U/mg Protein)	274.8 ± 19.74	105.5 ± 10.53 ***	247.1 ± 13.03 ^###^	187.8 ± 12.93 ^#^	233.51 ± 20.12 ^###^
CAT (n moles/min/mg protein)	287.2 ± 20.63	97.34 ± 9.14 ***	251.02 ± 23.05 ^###^	191.05 ± 12.32 ^#^	243.37 ± 17.18 ^###^
GR (n moles/min/mg protein)	232.6 ± 21.02	151.7 ± 15.01 ***	219.2 ± 21.2 ^###^	217.8 ± 11.54 ^##^	210.60 ± 19.3 ^##^
GSH (n mol/mg Protein)	292.8 ± 16.32	119.64 ± 11.8 ***	277.98 ± 18.0 ^###^	141.37 ± 13.1 ^#^	219.63 ± 13.7 ^##^

Results are representative of mean ± SE of eight mice per group. Values are significantly different in LiCl and pilocarpine group (*** *p* < 0.001) as compared to control group. Results that we got are significantly different from LiCl and pilocarpine group as the main comparison is with LiCl and pilocarpine group only (^#^ *p* < 0.05, ^##^ *p* < 0.01 and ^###^ *p* < 0.001). Group I: Normal saline (10 mL/kg b.w.), Group II: LiCl (3 mEq/kg b.w.) + pilocarpine (30 mg/kg b.w.), Group III: Sodium Valproate (300 mg/kg b.w.) + LiCl (3 mEq/kg b.w.) + pilocarpine (30 mg/kg b.w.), Group IV: zingerone (25 mg/kg b.w.) + LiCl (3 mEq/kg b.w.) + pilocarpine (30 mg/kg b.w.), Group V: zingerone (50 mg/kg b.w.) + LiCl (3 mEq/kg b.w.) + pilocarpine (30 mg/kg b.w.).

**Table 3:** Zingerone treatment effects on inflammatory markers on LiCl-and-pilocarpine-induced SE in experimental mice.

Inflammatory Markers	Group I	Group II	Group III	Group IV	Group V
NFk-B (pg/mL)	732.03 ± 53.4	1643.0 ± 136.7 ***	893.02 ± 67.0 ^###^	1342.5 ± 92.7 ^#^	922.11 ± 87.1 ^###^
TNF-α (pg/mL)	244.82 ± 19.4	765.62 ± 44.2 ***	269.05 ± 20.4 ^###^	517.81 ± 31.6 ^##^	288.53 ± 19.1 ^###^
IL-6 (pg/mL)	823.26 ± 47.2	1694.0 ± 140.2 ***	946.03 ± 40.2 ^###^	1440.5 ± 87.4 ^###^	997.16 ± 73.2 ^##^
IL-1β (pg/mL)	739.10 ± 51.8	1828.9 ± 121.8 ***	855.45 ± 74.0 ^###^	1514.1 ± 103.6 ^#^	893.73 ± 67.9 ^###^

Results are representative of mean ± SE of eight mice per group. Values are significantly different in LiCl and pilocarpine group (*** *p* < 0.001) as compared to control group. The results that we obtained are significantly different from LiCl and pilocarpine group as the main comparison is with LiCl and pilocarpine group only (^#^ *p* < 0.05, ^##^ *p* < 0.01 and ^###^ *p* < 0.001). Group I: Normal saline (10 mL/kg b.w.), Group II: LiCl (3 mEq/kg b.w.) + pilocarpine (30 mg/kg b.w.), Group III: Sodium valproate (300 mg/kg b.w.) + LiCl (3 mEq/kg b.w.) + pilocarpine (30 mg/kg b.w.), Group IV: zingerone (25 mg/kg b.w.) + LiCl (3 mEq/kg b.w.) + pilocarpine (30 mg/kg b.w.), Group V: zingerone (50 mg/kg b.w.) + LiCl (3 mEq/kg b.w.) + pilocarpine (30 mg/kg b.w.).

## Data Availability

The data presented in this study are available on request from the corresponding author.

## References

[1] (2019). World Health Organization (WHO). https://www.who.int/health-topics/epilepsy#tab=tab_1.

[2] Stafstrom C.E., Carmant L. (2015). Seizures and epilepsy: An overview for neuroscientists. Cold Spring Harb. Perspect. Med..

[3] Trinka E., Höfler J., Leitinger M., Brigo F. (2015). Pharmacotherapy for Status Epilepticus. Drugs.

[4] Zhao F., Kang H., You L., Rastogi P., Venkatesh D., Chandra M. (2014). Neuropsychological deficits in temporal lobe epilepsy: A comprehensive review. Ann. Indian Acad. Neurol..

[5] Trinka E., Cock H., Hesdorffer D., Rossetti A.O., Scheffer I.E., Shinnar S., Shorvon S., Lowenstein D.H. (2015). A definition and classification of status epilepticus--Report of the ILAE Task Force on Classification of Status Epilepticus. Epilepsia.

[6] Kinjo E.R., Higa G.S., Santos B.A., de Sousa E., Damico M.V., Walter L.T., Morya E., Valle A.C., Britto L.R., Kihara A.H. (2016). Pilocarpine-induced seizures trigger differential regulation of microRNA-stability related genes in rat hippocampal neurons. Sci. Rep..

[7] Deng X., Wang M., Hu S., Feng Y., Shao Y., Xie Y., Wu M., Chen Y., Shi X. (2019). The Neuroprotective Effect of Astaxanthin on Pilocarpine-Induced Status Epilepticus in Rats. Front. Cell. Neurosci..

[8] Shin E.J., Jeong J.H., Chung Y.H., Kim W.K., Ko K.H., Bach J.H., Hong J.S., Yoneda Y., Kim H.C. (2011). Role of oxidative stress in epileptic seizures. Neurochem. Int..

[9] Wang A., Si Z., Li X., Lu L., Pan Y., Liu J. (2019). FK506 Attenuated Pilocarpine-Induced Epilepsy by Reducing Inflammation in Rats. Front. Neurol..

[10] Wang Z., Zhou L., An D., Xu W., Wu C., Sha S., Li Y., Zhu Y., Chen A., Du Y. (2019). TRPV4-induced inflammatory response is involved in neuronal death in pilocarpine model of temporal lobe epilepsy in mice. Cell Death Dis..

[11] Rehman M.U., Wali A.F., Ahmad A., Shakeel S., Rasool S., Ali R., Rashid S.M., Madkhali H., Ganaie M.A., Khan R. (2019). Neuroprotective Strategies for Neurological Disorders by Natural Products: An update. Curr. Neuropharmacol..

[12] Safhi M.M. (2018). Nephroprotective Effect of Zingerone against CCl_4_-Induced Renal Toxicity in Swiss Albino Mice: Molecular Mechanism. Oxi. Med. Cell. Longev..

[13] Ganaie M.A., Al Saeedan A., Madhkali H., Jan B.L., Khatlani T., Sheikh I.A., Rehman M.U., Wani K. (2019). Chemopreventive efficacy zingerone (4-[4-hydroxy-3-methylphenyl] butan-2-one in experimental colon carcinogenesis in Wistar rats. Environ. Toxicol..

[14] Rehman M.U., Rashid S.M., Rasool S., Shakeel S., Ahmad B., Ahmad S.B., Madkhali H., Ganaie M.A., Majid S., Bhat S.A. (2019). Zingerone (4-(4-hydroxy-3-methylphenyl)butan-2-one) ameliorates renal function via controlling oxidative burst and inflammation in experimental diabetic nephropathy. Arch. Physiol. Biochem..

[15] Min G., Ku S.K., Lee T., Bae J.S. (2018). Suppressive effects of zingerone on TGFBIp-mediated septic responses. Arch. Pharmacol. Res..

[16] Kim C.Y., Seo Y., Lee C., Park G.H., Jang J.H. (2018). Neuroprotective Effect and Molecular Mechanism of [6]-Gingerol against Scopolamine-Induced Amnesia in C57BL/6 Mice. Evid. Based Complement. Altern. Med. eCAM.

[17] Safhi M.M. (2015). Zingerone protects the tellurium toxicity in the brain mitochondria of rats. Metabolomics.

[18] Li L.L., Cui Y., Guo X.H., Ma K., Tian P., Feng J., Wang J.M. (2019). Pharmacokinetics and Tissue Distribution of Gingerols and Shogaols from Ginger (Zingiber officinale Rosc.) in Rats by UPLC⁻Q-Exactive⁻HRMS. Molecules..

[19] Kabuto H., Yamanushi T.T. (2011). Effects of zingerone [4-(4-hydroxy-3-methoxyphenyl)-2-butanone] and eugenol [2-methoxy-4-(2-propenyl)phenol] on the pathological progress in the 6-hydroxydopamine-induced Parkinson’s disease mouse model. Neurochem. Res..

[20] Vaibhav K., Shrivastava P., Tabassum R., Khan A., Javed H., Ahmed M.E., Islam F., Safhi M.M., Islam F. (2013). Delayed administration of zingerone mitigates the behavioral and histological alteration via repression of oxidative stress and intrinsic programmed cell death in focal transient ischemic rats. Pharmacol. Biochem. Behavior..

[21] Al Kury L.T., Mahgoub M., Howarth F.C., Oz M. (2018). Natural Negative Allosteric Modulators of 5-HT₃ Receptors. Molecules.

[22] Venkatanarayana N. (2013). Evaluation of anticonvulsant activity of ethanolic extract of *Zingiber officinale* in Swiss albino rats. J. Chem. Pharm. Res..

[23] Walker M.C. (2018). Pathophysiology of status epilepticus. Neurosci. Lett..

[24] Trinka E., Brigo F., Shorvon S. (2016). Recent advances in status epilepticus. Curr. Opin. Neurol..

[25] Soliman A.F., Anees L.M., Ibrahim D.M. (2018). Cardioprotective effect of zingerone against oxidative stress, inflammation, and apoptosis induced by cisplatin or gamma radiation in rats. Naunyn Schmiedeberg Arch. Pharmacol..

[26] Phelan K.D., Shwe U.T., Williams D.K., Greenfield L.J., Zheng F. (2015). Pilocarpine-induced status epilepticus in mice: A comparison of spectral analysis of electroencephalogram and behavioral grading using the Racine scale. Epilepsy Res..

[27] Greenfield L.J. (2013). Molecular mechanisms of antiseizure drug activity at GABAA receptors. Seizure.

[28] Yue H.Y., Jiang C.Y., Fujita T., Kumamoto E. (2013). Zingerone enhances glutamatergic spontaneous excitatory transmission by activating TRPA1 but not TRPV1 channels in the adult rat substantia gelatinosa. J. Neurophysiol..

[29] Castle N.A. (1992). Differential inhibition of potassium currents in rat ventricular myocytes by capsaicin. Cardiovasc. Res..

[30] Erdélyi L. (1999). Guaiacol and vanilloid compounds modulate the A-type potassium currents in molluscan neurons. Acta Biol. Hung..

[31] Kim J.N., Kim H.J., Kim I., Kim Y.T., Kim B.J. (2018). The Mechanism of Action of Zingerone in the Pacemaker Potentials of Interstitial Cells of Cajal Isolated from Murine Small Intestine. Cell. Physiol. Biochem. Int. J. Exp. Cell. Physiol. Biochem. Pharmacol..

[32] George K., Thomas N.S., Malathi R. (2019). Modulatory Effect of Selected Dietary Phytochemicals on Delayed Rectifier K+ Current in Human Prostate Cancer Cells. J. Membr. Biol..

[33] Marafiga J.R., Pasquetti M.V., Calcagnotto M.E. (2020). GABAergic interneurons in epilepsy: More than a simple change in inhibition. Epilepsy Behav..

[34] Hosseini A., Mirazi N. (2014). Acute administration of ginger (Zingiber officinale rhizomes) extract on timed intravenous pentylenetetrazol infusion seizure model in mice. Epilepsy Res..

[35] Lerche H., Shah M., Beck H., Noebels J., Johnston D., Vincent A. (2013). Ion channels in genetic and acquired forms of epilepsy. J. Physiol..

[36] Power K.N., Gramstad A., Gilhus N.E., Hufthammer K.O., Engelsen B.A. (2018). Cognitive dysfunction after generalized tonic-clonic status epilepticus in adults. Acta. Neurol. Scand..

[37] Martinc B., Grabnar I., Vovk T. (2014). Antioxidants as a preventive treatment for epileptic process: A review of the current status. Curr. Neuropharmacol..

[38] Kandeda A.K., Taiwe G.S., Moto F.C.O., Ngoupaye G.T., Nkantchoua G.C.N., Njapdounke J.S.K., Omam J.P.O., Pale S., Kouemou N., Bum E.N. (2017). Antiepileptogenic and Neuroprotective Effects of Pergularia daemia on Pilocarpine Model of Epilepsy. Front. Pharmacol..

[39] Pearson-Smith J.N., Patel M. (2017). Metabolic dysfunction and oxidative stress in epilepsy. Int. J. Mol. Sci..

[40] Rajan I., Narayanan N., Rabindran R., Jayasree P.R., Kumar P.R.M. (2013). Zingerone protects against stannous chloride-induced and hydrogen peroxide-induced oxidative DNA damage in vitro. Biol. Trace. Elem. Res..

[41] Diniz T.C., Silva J.C., de Lima-Saraiva S.R., Ribeiro F.P., Pacheco A.G., de Freitas R.M., Quintans-Júnior L.J., Quintans J., Mendes R.L., Almeida J.R. (2015). The role of flavonoids on oxidative stress in epilepsy. Oxi. Med. Cell. Longev..

[42] Mani V., Arivalagan S., Siddique A.I., Namasivayam N. (2016). Antioxidant and anti-inflammatory role of zingerone in ethanol-induced hepatotoxicity. Mol. Cell. Biochem..

[43] Carmona-Aparicio L., Zavala-Tecuapetla C., González-Trujano M.E., Sampieri A.I., Montesinos-Correa H., Granados-Rojas L., Floriano-Sánchez E., Coballase-Urrutía E., Cárdenas-Rodríguez N. (2016). Status epilepticus: Using antioxidant agents as alternative therapies. Exp. Ther. Med..

[44] Amin I., Hussain I., Rehman M.U., Mir B.A., Ganaie S.A., Ahmad S.B., Mir M., Shanaz S., Muzamil S., Arafah A. (2020). Zingerone prevents lead-induced toxicity in liver and kidney tissues by regulating the oxidative damage in Wistar rats. J. Food Biochem..

[45] McElroy P.B., Liang L.P., Day B.J., Patel M. (2017). Scavenging reactive oxygen species inhibits status epilepticus-induced neuroinflammation. Exp. Neurol..

[46] da Silva A.P.D.S., Lopes J.S., Vieira P.D.S., Pinheiro E.E., da Silva M.L.D.G., Silva Filho J.C.C., da Costa Júnior J.S., David J.M., de Freitas R.M. (2014). Behavioral and neurochemical studies in mice pretreated with garcinielliptone FC in pilocarpine-induced seizures. Pharmacol. Biochem. Behav..

[47] Gnatek Y., Zimmerman G., Goll Y., Najami N., Soreq H., Friedman A. (2012). Acetylcholinesterase loosens the brain’s cholinergic anti-inflammatory response and promotes epileptogenesis. Front. Mol. Neurosci..

[48] Aronica E., Bauer S., Bozzi Y., Caleo M., Dingledine R., Gorter J.A., Henshall D.C., Kaufer D., Koh S., Löscher W. (2017). Neuroinflammatory targets and treatments for epilepsy validated in experimental models. Epilepsia.

[49] Ahmad B., Rehman M.U., Amin I., Mir M., Ahmad S.B., Farooq A., Muzamil S., Hussain I., Masoodi M., Fatima B. (2018). Zingerone (4-(4-hydroxy-3-methylphenyl) butan-2-one) protects against alloxan-induced diabetes via alleviation of oxidative stress and inflammation: Probable role of NF-kB activation. Saud. Pharm. J..

[50] Shimada T., Takemiya T., Sugiura H., Yamagata K. (2014). Role of inflammatory mediators in the pathogenesis of epilepsy. Mediators. Inflamm..

[51] Wali A.F., Rehman M.U., Raish M., Kazi M., Rao P., Alnemer O., Ahmad P., Ahmad A. (2020). Zingerone [4-(3-Methoxy-4-hydroxyphenyl)-butan-2] Attenuates Lipopolysaccharide-Induced Inflammation and Protects Rats from Sepsis Associated Multi Organ Damage. Molecules.

[52] Borges K., Gearing M., McDermott D.L., Smith A.B., Almonte A.G., Wainer B.H., Dingledine R. (2003). Neuronal and glial pathological changes during epileptogenesis in the mouse pilocarpine model. Exp. Neurol..

[53] Ahmad B., Rehman M.U., Amin I., Arif A., Rasool S., Bhat S.A., Afzal I., Hussain I., Bilal S., Mir M.U.R. (2015). A Review on Pharmacological Properties of Zingerone (4-(4-Hydroxy-3-methoxyphenyl)-2-butanone). Sci. World J..

[54] Džoljić E., Grbatinić I., Kostić V. (2015). Why is nitric oxide important for our brain?. Funct. Neurol..

[55] Mir B., Amin I., Rehman M.U., Bhat R., Ali A., Baba O.K., Fatima B., Ali R., Ahmad S.B., Muzamil S. (2018). Chemoprotective potential of zingerone (vanillyl acetone) in cyclophosphamide-induced hepatic toxicity. Pharmacogn. Mag..

[56] Vasconcelos Rios E.R., Moura Rocha N.F., Rodrigues Carvalho A.M., Freire Vasconcelos L., Leite Dias M., de Carvalho Lima C.N., Soares Lopes K., Cavalcante Melo F.H., de França Fonteles M.M. (2013). Involvement of the nitric oxide/cyclic guanylate monophosphate pathway in the pilocarpine-induced seizure model in mice. Pharmacology.

[57] Zhang Y., Seeburg D.P., Pulli B., Wojtkiewicz G.R., Bure L., Atkinson W., Schob S., Iwamoto Y., Ali M., Zhang W. (2016). Myeloperoxidase Nuclear Imaging for Epileptogenesis. Radiology.

[58] Rehman M.U., Ahmad B., Arif A., Rasool S., Farooq A., Razzaq R., Bhat S.A., Bashir S., Shabir O., Amin I. (2015). Zingerone protects against cisplatin-induced oxidative damage in the jejunum of Wistar rats. Orient. Pharm. Exp. Med..

[59] Marisela M.A., Concepción N.R., Daniel J.R., Erika R.M., Petra Y.G. (2014). Oxidative Stress Associated with Neuronal Apoptosis in Experimental Models of Epilepsy. Oxidative Med. Cell. Longev..

[60] Engel T., Henshall D.C. (2009). Apoptosis, Bcl-2 family proteins and caspases: The ABCs of seizure-damage and epileptogenesis?. Int. J. Physiol. Pathophysiol. Pharmacol..

[61] Mao X.Y., Zhou H.H., Jin W.L. (2019). Redox-Related Neuronal Death and Crosstalk as Drug Targets: Focus on Epilepsy. Front. Neurosci..

[62] Folbergrová J., Kunz W.S. (2012). Mitochondrial dysfunction in epilepsy. Mitochondrion.

[63] Cho I., Cho Y.J., Kim H.W., Heo K., Lee B.I., Kim W.J. (2014). Effect of Androsterone after Pilocarpine-induced Status Epilepticus in Mice. J. Epilepsy Res..

[64] Ahmed Juvale I.I., Che Has A.T. (2020). The evolution of the pilocarpine animal model of status epilepticus. Heliyon.

[65] Kandemir F.M., Yildirim S., Caglayan C., Kucukler S., Eser G. (2019). Protective effects of zingerone on cisplatin-induced nephrotoxicity in female rats. Environ. Sci. Pollut. Res..

[66] Kaygusuzoglu E., Caglayan C., Kandemir F.M., Yıldırım S., Kucukler S., Kılınc M.A., Saglam Y.S. (2018). Zingerone ameliorates cisplatin-induced ovarian and uterine toxicity via suppression of sex hormone imbalances, oxidative stress, inflammation and apoptosis in female wistar rats. Biomed. Pharmacother..

[67] Bilal A., Insha A., Towseef A., Muneeb U.R., Showkat A., Saiema R., Ahmad A., Adil F., Showkeen M., Ishraq H. (2016). Zingerone (4-(4-hydroxy-3-methoxyphenyl)-2-butanone) Protects Against Acetaminophen Induced Hepatotoxicity in Wistar Rats via Alleviation of Oxidative Stress and Inflammation. Asian J. Anim. Vet. Adv..

[68] Shakeel S., Rehman M.U., Tabassum N., Amin U., Mir R. (2017). Effect of Naringenin (A naturally occurring flavanone) Against Pilocarpine-induced Status Epilepticus and Oxidative Stress in Mice. Pharmacogn. Mag..

[69] Vorhees C.V., Williams M.T. (2006). Morris water maze: Procedures for assessing spatial and related forms of learning and memory. Nat. Protocol..

[70] Wright J.R., Colby H.D., Miles P.R. (1981). Cytosolic factors which affect microsomal lipid peroxidation in lung and liver. Arch. Biochem. Biophys..

[71] Marklund S., Marklund G. (1974). Involvement of the superoxide anion radical in the autoxidation of pyrogallol and a convenient assay for superoxide dismutase. Eur. J. Biochem..

[72] Claiborne A., Greenwald R.A. (1985). Catalase activity. CRC Handbook of Methods in Oxygen Radical Research.

[73] Rashid S., Ali N., Nafees S., Hasan S.K., Sultana S. (2014). Mitigation of 5-Fluorouracil induced renal toxicity by chrysin via targeting oxidative stress and apoptosis in wistar rats. Food. Chem. Toxicol..

[74] Ellman G.L., Courtney K.D., Andres V., Featherstone R.M. (1961). A new and rapid colorimetric determination of acetylcholinesterase activity. Biochem. Pharmacol..

[75] Bradley P.P., Priebat D.A., Christensen R.D., Rothstein G. (1982). Measurement of cutaneous inflammation: Estimation of neutrophil content with an enzyme marker. J. Investig. Dermatol..

[76] Liua C.M., Mab J.Q., Suna J.Q. (2010). Quercetin protects the rat kidney against oxidative stress-mediated DNA damage and apoptosis induced by lead. Environ. Toxicol. Pharmacol..

[77] Green L.C., Wagner D.A., Glogowski J., Skipper P.L., Wishnok J.S., Tannenbaum S.R. (1982). Analysis of nitrate, nitrite, and [15N] nitrate in biological fluids. Anal. Biochem..

[78] Lowry O.H., Rosebrough N.J., Farr A.L. (1951). Protein measurement with the Folin Phenol reagent. J. Biol. Chem..

[79] Rashid S., Nafees S., Vafa A., Afzal S.M., Ali N., Rehman M.U., Hasan S.K., Siddiqi A., Barnwal P., Majed F. (2016). Inhibition of precancerous lesions development in kidneys by chrysin via regulating hyperproliferation, inflammation and apoptosis at pre clinical stage. Arch. Biochem. Biophys..

[80] Szilágyi T., Orbán-Kis K., Horváth E., Metz J., Pap Z., Pávai Z. (2011). Morphological identification of neuron types in the rat hippocampus. Romanian J. Morphol. Embryol. Rev. Roum. Morphol. Embryol..

